# Emerging RNA-centric technologies to probe RNA-protein interactions: importance in decoding the life cycle of positive sense single strand RNA viruses and antiviral discovery

**DOI:** 10.3389/fcimb.2025.1580337

**Published:** 2025-05-21

**Authors:** Sreemoyee Ghosh, Shivam Kumar, Rohit Verma, Shabnam Ansari, Samrat Chatterjee, Milan Surjit

**Affiliations:** ^1^ Virology Laboratory, Centre for Virus Research, Therapeutics and Vaccines, Translational Health Science and Technology Institute, NCR Biotech Science Cluster, Faridabad, Haryana, India; ^2^ Complex Analysis Group, Computational and Mathematical Biology Centre, Translational Health Science and Technology Institute, NCR Biotech Science Cluster, Faridabad, Haryana, India

**Keywords:** RNA-protein interactions, RNA binding protein, positive strand RNA viruses, RaPID assay, RAP-MS

## Abstract

Positive sense single strand RNA (+ssRNA) viruses are one of the evolutionary successful organisms and many of them pose a significant threat to human health. Diseases caused by +ssRNA viruses such as COVID-19, Flu and acute viral hepatitis are major public health concern worldwide. Therefore, a lot of research is focused at decoding the life cycle of +ssRNA viruses and develop specific antiviral therapeutics against them. Interaction of the viral RNA with virus-encoded proteins and host proteins drives the lifecycle and pathogenesis of +ssRNA viruses. Recent developments in computational and high-throughput omics-based experimental technologies offer the sensitivity and specificity for molecular characterization of these RNA-protein complexes. These are promising tools to revolutionize the field of +ssRNA virus research and pave the way for antiviral discovery. This review summarizes the current scientific resources available to characterize the RNA-protein interactome of +ssRNA viruses and provides an overview of the drug discovery pipeline for developing antivirals against pathogenic +ssRNA viruses.

## 1 Introduction

The central dogma of molecular biology signifies the importance of flow of genetic information from DNA to RNA to protein. Decades of research have further uncovered multiple layers of complex mechanisms by which biological systems accurately process the flow of information and maintain homeostasis. Such precision and specificity of the biological systems are mostly attributed to close interaction between different components of the system.

RNA and proteins are two fundamental components of living organisms, required for their survival and propagation. Ribosomal-RNA, transfer-RNA and messenger-RNA work in a coordinated fashion to generate proteins, which perform major cellular function to maintain homeostasis. Interaction between RNA and proteins (RNA-binding proteins, denoted as “RBPs” hereafter) plays a major role in mediating the function of both and such interactions are indispensable for many essential processes in living organisms. RBPs serve diverse cellular functions: for example, RBP-RNA interacts to form the ribonucleoprotein particles (RNPs), dynamic complexes, involved in different steps of gene expression, intracellular trafficking of RNA, decay of RNA and control of protein turnover etc (Dreyfuss et al., 2002; Gerstberger et al., 2014). The RBPs function by synergistically interacting with structurally well-defined binding domains. Although these domains are limited in number, they are tailored to perform specific function (Lunde et al., 2007). The major RBP binding domains with over 100 PDB structures are Zinc Finger, Helicase, RNA Recognition Motif, PUA domain, and KH domain (Corley et al., 2020). RBPs are consistent with their frequent housekeeping roles, widely distributed across tissues, and more evolutionarily conserved than standard regulators like transcription factors (Gerstberger et al., 2014).

In addition to endogenous cellular regulations, RBPs play a pivotal role in determining the fate of pathogens, such as viruses, within our bodies. Pathogenic +ssRNA viruses are a major human health concern. Owing to simple organization and high mutation rate of their genome, they generate a number of distinct variants in a short span of time, making it more difficult to control their spread. Notably, the central dogma of flow of genetic information in +ssRNA viruses rely only on two components, that is, from RNA to protein. Viral RNA serves as the genetic material and with the help of virus-encoded proteins and host proteins, it plays a central role in transmission, spread and maintenance of genomic integrity of the virus. Knowledge gained from research on many +ssRNA viruses suggest that specific and spatio-temporally controlled RNA-protein interactions among viral RNA and proteins as well as viral RNA/proteins and host RNA/proteins enable these viruses to hijack the host cellular machineries in order to survive and proliferate inside the host and maintain their genomic integrity through generations (Nagy and Pogany, 2012; Robinson et al., 2018). Therefore, molecular dissection of these RNA-protein interactions is key to understanding the mechanistic details of survival and spread of the pathogenic +ssRNA viruses as well as designing specific antiviral therapeutics against them.

Due to the unstable nature and crucial role of secondary and tertiary structures of RNA in dictating its function, it is not easy to characterize RNA-protein interactions. However, with the development of more sensitive proteomics techniques and computational methods, it is now possible to construct the RNA-protein interactome of +ssRNA viruses. RNA-protein interactome of few +ssRNA viruses such as SARS-CoV-2 and Zika virus have been generated, which helped in understanding the life cycle of the virus and identification of putative antiviral targets (Flynn et al., 2021; Kamel et al., 2021a; Schmidt et al., 2021; Verma et al., 2021; Zhang et al., 2022). In this review, we focus on available RNA-centric techniques to construct the RNA-protein interactome and discuss the functional significance of the data in understanding the life cycle of +ssRNA viruses and antiviral target discovery.

## 2 RNA-protein interactions help the +ssRNA viruses escape the host innate immune response and complete their life cycle

### 2.1 RNA-protein interactions in viral evasion of the host innate immune response

**Figure 1:**
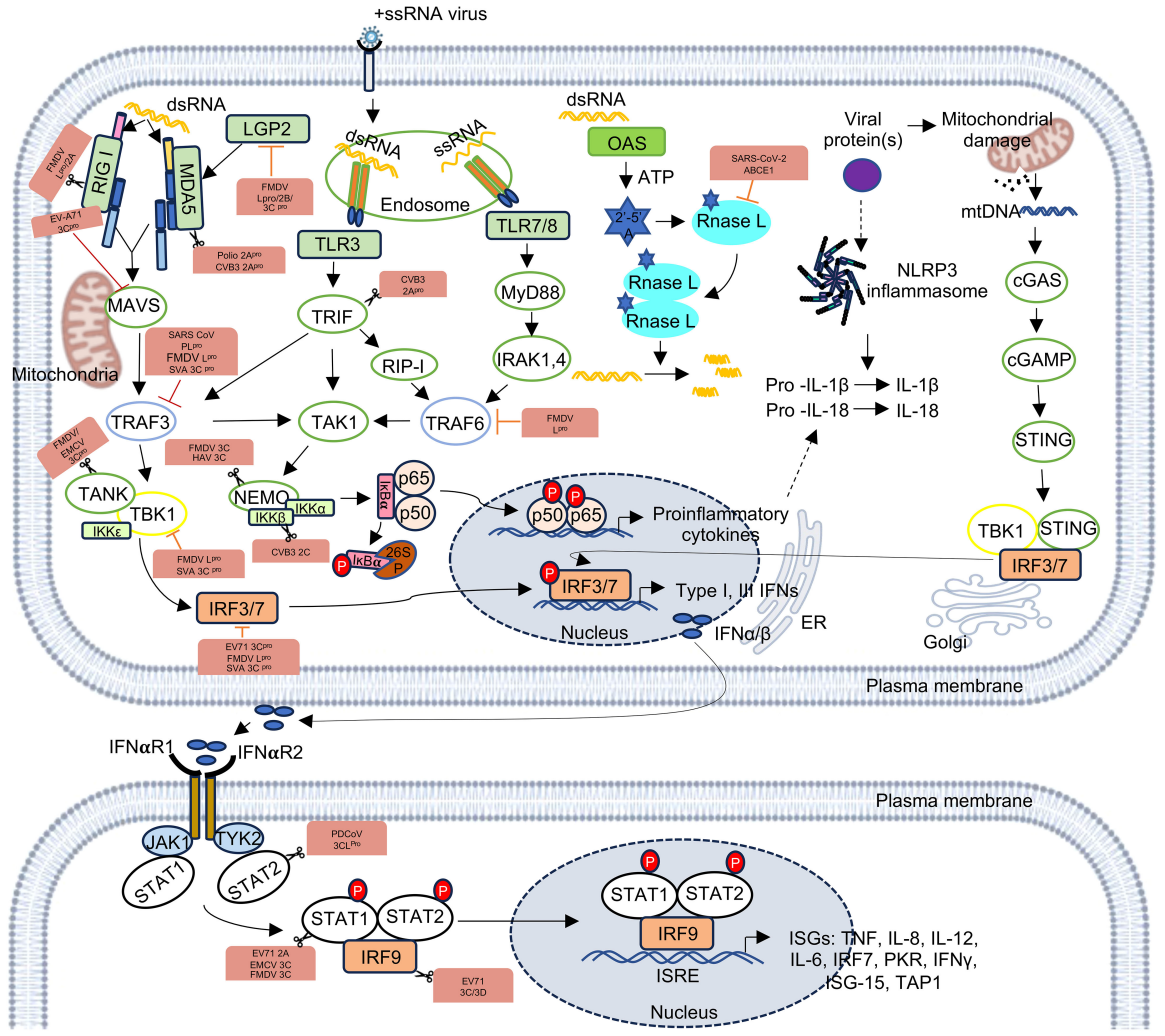
Recognition of +ssRNA viruses by the host innate immune pathways and generation of antiviral response. RIG-I and MDA5 recognize dsRNA, TLR3 and TLR7/8 sense dsRNA and ssRNA, respectively, and activate the indicated pathways to express type I and type III interferons and proinflammatory cytokines. dsRNA also activates RNase L, which cleaves the former. Viral proteins can damage mitochondria and/or activate the inflammasome. RIG-I, Retinoic acid-inducible gene; MDA5, Melanoma differentiation-associated gene 5; LGP2, Laboratory of genetics and physiology 2; MAVS, Mitochondrial antiviral signaling protein; TRAF3, TNF receptor associated factor 3; TBK1, TANK-binding kinase 1; IKKϵ, IκB kinase ϵ; IRF3/7, Interferon regulatory factor 3 or 7; TLR3, toll-like receptor 3; TLR7/8, toll-like receptor 7 or 8; TRIF, TIR-domain containing adaptor inducing interferon-β; RIP-1, receptor-interacting protein 1; TRAF6, TNF receptor associated factor 6; TAK1, TGFβ-activated kinase 1; IKKα/β, IκB kinase α/β; MyD88, Myeloid differentiation primary response 88; IRAK1,4, interleukin-1 receptor-associated kinase 1,4; OAS, oligoadenylate synthetase; 2’-5’ A, 2’-5’ oligoadenylate; NLRP3, NOD-like receptor thermal protein domain associated protein 3; cGAS, cyclic GMP-AMP synthase; cGAMP, cyclic GMP-AMP; STING, Stimulator of interferon genes,IL-1β, Interleukin-1β; IFN-α/β, interferon α/β; IFNαR1/2, interferon α/β receptor 1/2; JAK1, janus kinase 1; TYK2, tyrosine kinase 2; STAT1/2, signal transducer and activator of transcription ½; IRF9, interferon regulatory factor 9; ISRE, interferon stimulated response element; ISGs, interferon stimulatory genes. The figure is made in Microsoft PowerPoint and BioRender.

Host innate immune effectors differentiate between self and non-self RNAs. After entry of an +ssRNA virus into the host cell, viral RNA is released from the capsid, which may be recognized by the host antiviral immune effectors such as Toll like receptor 7/8 (TLR7/8), 2′-5′-oligoadenylate synthetase (OAS)/RNase L and targeted for degradation (**Figure 1**) (Chan and Gack, 2016). Further, during replication of the viral genome, double-strand RNA is generated, which is recognized by host antiviral immune effectors such as Toll-like receptors (TLRs) and RIG-I-like receptors (RLRs). Among the TLRs, TLR3 and RLR family proteins like, retinoic acid inducible gene-I (RIG-I), melanoma differentiation-associated gene 5 (MDA5) and laboratory of genetics and physiology 2 (LGP2) channelize the viral RNA for degradation (**Figure 1**) (Ma and Suthar, 2015; Chan and Gack, 2016). In many cases, +ssRNA virus infection also causes mitochondrial damage, resulting in the release of mitochondrial DNA, which is sensed by the cyclic GMP-AMP (cGMP) synthase (cGAS), leading to the induction of type I interferon and interferon-stimulated genes (ISGs), thereby mounting a strong antiviral response. RNA viruses also activate NOD-like receptor thermal protein domain associated protein 3 (NLRP3), activating inflammasomes and/or pyroptosis (Choudhury et al., 2021). Viral RNA may also modulate cellular autophagy machinery and components of the stress granule, RNA granule or P bodies due to their link with the host’s innate immune response (White and Lloyd, 2012; Tsai and Lloyd, 2014). Interaction between the viral RNA and host proteins mediate the above-mentioned processes. For example, RNA-protein interactome of the SARS-CoV-2 5’- and 3’-UTR regions identified DDX24 and ABCE1 as interaction partners of the viral 3’-UTR and 5’-UTR, respectively (Verma et al., 2021). DDX24 associates with RNA and negatively regulates RIG-I-like receptor signaling, inhibiting the host antiviral response (Ma et al., 2013). ABCE1 (RNase L inhibitor) inhibits the activity of RNase L, which is activated by the host in response to RNA virus infection or interferon alpha/beta (IFN-α/β) stimulation (Tian et al., 2012). Active RNase L cleaves the viral RNA, which is prevented in the presence of ABCE1. Hence, DDX24-3’-UTR and ABCE1-5’-UTR interactions appear to be immune evasion strategies of the SARS-CoV-2. A phylogenetically conserved RNA structure within the 3C region of Polio virus ORF actively inhibits the endoribonuclease activity of RNase L (Han et al., 2007). RNA-protein interactome of the SARS-CoV-2 5’- and 3’-UTR regions also identified the antiviral role of LAMP2a, which is the receptor for chaperone-mediated autophagy (Verma et al., 2021). DENV-2 PR-2B sfRNA (sub-genomic RNA fragments) interacts with TRIM25, interferes with its deubiquitylation and inhibits RIG-I signaling (Manokaran et al., 2015). DENV-2 non-coding sfRNA interacts with G3BP1, G3BP2 and CAPRIN1 and inactivates them to suppress the expression of ISGs (Bidet et al., 2017). N^6^-methyladenosine (m6A) modification of HCV and SARS-CoV-2 RNA helps them in evading recognition by RIG-I (Kim et al., 2020; Li et al., 2021). MRM2/FTSJ2, a mitochondrial 2’-O-methyltransferase interacts with the SARS-CoV-2 RNA, which might shield the viral RNA from recognition by MDA5 (Flynn et al., 2021). NSP15 of coronaviruses (CoVs) encode endoribonuclease EndoU, which cleaves the viral polyuridine sequence, inhibiting the activation of host immune sensors. The viral 5’-polyuridine from negative-sense viral RNA, termed PUN RNA is the product of polyA-templated RNA synthesis and is an MDA5-dependent pathogen-associated molecular pattern (PAMP) (Hackbart et al., 2020).

Notably, viral proteases also play a key role in inhibiting the host innate immune components. For example, Picornavirus 2A^pro^ disrupts MDA5-MAVS mediated antiviral innate immune response, Coxsackievirus B3 (CVB3) 2A^pro^ cleaves MDA5 and MAVS by caspase-proteasome independent pathway, while poliovirus (PV) 2A^pro^ cleaves MDA5 via caspase-proteosome dependent pathway, CVB3 2A^pro^ cleaves TRIF, thus antagonizing type-I and type-III interferon production (Lind et al., 2016). The RLR signaling pathway is disrupted by the 3C^pro^ of picornavirus. 3C^pro^ of EV-A71 binds to the N-terminal CARDs of RIG-I, inhibiting its interaction with MAVS, and thus disrupting activation of type-I IFN response and 3C^pro^ of encephalomyocarditis virus (EMCV) cleaves RIG-I *in vitro*, promoting its degradation by the caspase pathway (Papon et al., 2009; Lei et al., 2010). EMCV 3C^pro^ also disrupts the TANK–TBK1–IKKϵ–IRF3 complex by cleaving TANK, thus decreasing type‐I IFN production (Huang et al., 2017). FMDV 3C^pro^ disrupts NF‐κB and IRF3 signaling pathway by cleaving the C-terminal zinc finger domain of IKKγ (Wang et al., 2012). FMDV 3C^pro^ and 2B proteins inhibit LGP2 expression (Zhu et al., 2017).

Proteases of coronaviridae also interferes with innate immune response (Lei and Hilgenfeld, 2017). SARS-CoV PL^pro^ reduces the ubiquitination of STING, TRAF3 and TBK1, thus prohibiting their activation (Chen et al., 2014). It also stabilizes the IκBα and inhibits NF‐κB signaling pathway (Frieman et al., 2009). 3CL^pro^ (Also known as M^pro^) of porcine deltacoronavirus (PDCoV) and porcine epidemic diarrhea virus (PEDV) cleaves IKKγ, thereby abrogating NF-κB signaling (Wang et al., 2016; Zhu et al., 2017a). 3CL^pro^ of PDCoV cleaves STAT2, 2A of EV71, 3C of EMCV, and 3C of FMDV cleave STAT1, and 3C, 3D proteases of EV71 cleave IRF9 and disrupt the JAK-STAT pathway (Du et al., 2014; Wang et al., 2015; Huang et al., 2017; Zhu et al., 2017b). Further, leader protease (L^pro^), found in many picornaviruses targets multiple host innate immune factors to promote survival of the virus. The FMDV-L^pro^ cleaves LGP2, inhibiting the type I IFN response (Rodríguez Pulido et al., 2018). FMDV-L^pro^ also induces the degradation of p65/RelA subunit of NF-κB and decreases the expression of IRF3 and IRF7, leading to inhibition of the NF-κB activity and IFN-α/β expression, respectively (de Los Santos et al., 2007; Wang et al., 2010). A shorter form of FMDV-L^pro^, known as Lb^pro^, inhibits the ubiquitination of RIG-I, TBK1, TRAF6, and TRAF3, thereby inhibiting the secretion of type I IFNs (Wang et al., 2011). The L^pro^ of Theiler’s murine encephalomyelitis virus (TMEV) and Mengovirus inhibits IRF3 activity and blocks IFN-β transcription (Hato et al., 2007; Stavrou et al., 2010). Mengovirus-L^pro^ also inhibits NF-κB activity, leading to inhibition of IFN-α/β expression in virus-infected cells (Zoll et al., 2002).

### 2.2 RNA-protein interactions drive the progress through different stages in the life cycle of +ssRNA viruses

**Figure 2:**
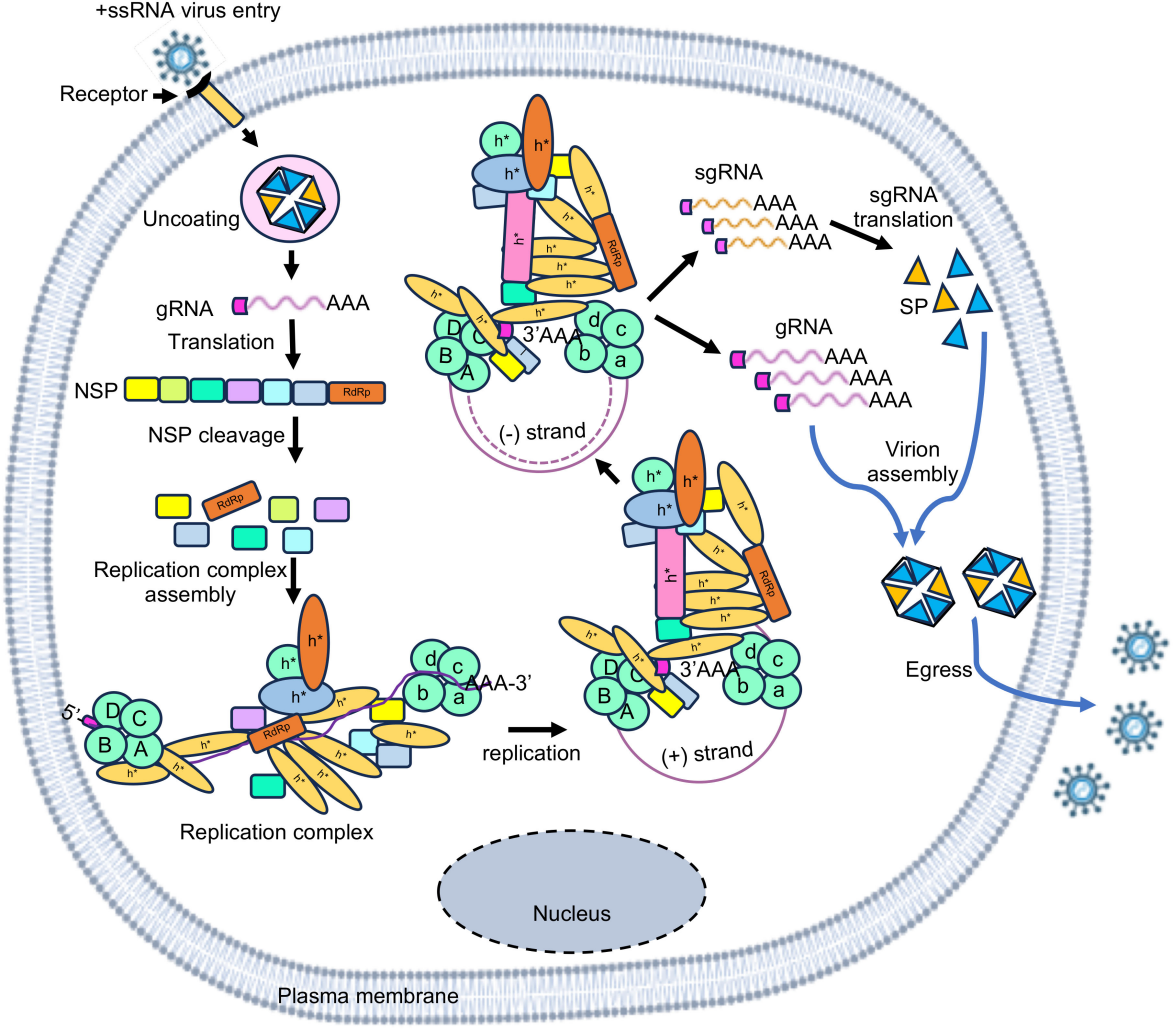
Simplified illustration of life cycle of +ssRNA viruses. The RNA virus life cycle has four major steps- entry, replication, assembly, and egress. After entry into the host and uncoating of the viral capsid, viral genomic RNA is translated to produce the non-structural polyprotein (NSP), which is subsequently processed into individual subunits. Viral RdRp assembles a RNA-protein complex, which interacts with the RNA-protein complexes assembled at 5’- and 3’- termini of the viral genomic RNA to form the viral replication complex. Viral genomic RNA likely forms a closed loop structure during replication. Antisense strand (-) as well as sub-genomic (sg) and genomic (g) RNA strands are synthesized by replication. Sub-genomic RNA is translated to produce the structural proteins (SP) that assembles the viral capsid, which encapsulates the genomic RNA. Progeny virions are subsequently released outside. A B C D illustrate 5’-UTR-interacting host proteins; a b c d illustrate 3’-UTR-interacting host proteins; h* illustrate host proteins interacting with the RdRp-bound RNA-protein complex.

The life cycle of a +ssRNA virus starts with the entry of the virus into the host cell. Post uncoating, the viral genome is released to the cytoplasm, where it serves as the template for translation of the non-structural and/or structural polyprotein, followed by their cleavage through autolysis and/or with the help of virus-encoded and/or host proteases. Translation of proteins in +ssRNA viruses may be mediated via cap-dependent, cap-independent or a combination of both mechanisms. The presence of the 5’- end cap stabilizes the viral RNA and protects it from getting degraded by the host nucleases. The 5’-cap also enables cap-dependent translation of the viral RNA, using the host translation machinery. Both cap-dependent and cap-independent translation is driven by the interaction of viral genomic RNA with a temporally regulated complex of host translation factors. For example, RNA-protein interactome of the SARS-CoV-2-5’- and 3’-UTR RNAs show enrichment of host translation factors (Verma et al., 2021). Note that SARS-CoV-2 translation is a cap-dependent process. RNA-protein interactome of the Hepatitis E virus internal ribosome entry site (HEV-IRES), which drives cap-independent translation of the viral ORF4 protein, also shows enrichment of host translation factors (Kumar et al., 2023b). Poly(rC) binding proteins1 and 2 (also known as PCBP1 and PCBP2) enhance Polio virus translation by forming RNP complex with stem loop IV of the viral IRES (Blyn et al., 1996). The PTB-associated splicing factor (PSF) interacts with the cloverleaf structure in the IRES of coxsackievirus B3 (CVB3) and this interaction plays important role in viral translation (Dave et al., 2017). Another host protein, RNA helicase A (RHA) interacts with S fragment in the 5’-UTR of Foot-and-mouth disease virus (FMDV) RNA (Lawrence and Rieder, 2009). In coronaviruses, the cap and the poly (A) tail of the viral genomic RNA recruit initiation factor(s) that support the formation of a closed loop RNA conformation, which favors efficient translation initiation (**Figure 2**) (Walsh and Mohr, 2011; Lo et al., 2019; Stern-Ginossar et al., 2019; Sorokin et al., 2021).

#### 2.2.1 RNA-protein interaction during the replication of +ssRNA viruses

Replication of the viral genome is central to the life cycle of a virus, which generates multiple copies of the viral genome to assemble progeny viruses. In the case of +ssRNA viruses, viral genomic RNA acts as the template and with the help of viral RNA-dependent RNA polymerase (RdRp) and many other viral and host proteins, viral genome is copied. RdRp usually binds to the +ssRNA virus genome at the 3’-end. Multiple host factors bind to the genome at the 5’- and 3’-ends, leading to the assembly of a RNA-protein complex, which facilitates circularization of the genome and formation of negative-strand RNA, sub-genomic RNAs and positive-strand genomic RNA (**Figure 2**). For example, genome circularization is important for replication of Flaviviruses (Villordo and Gamarnik, 2009). Nucleocapsid (N) protein of the Bovine Coronavirus (BCoV) interacts with both 5’- and 3’-ends of the viral genome, resulting in circularization of the viral genome, which is important for the synthesis of the negative strand RNA (Lo et al., 2019). Analysis of RNA-protein interactome of the SARS-CoV-2-5’- and 3’-UTR RNAs suggests PPI-mediated bridging of the 5’- and 3’- ends of the viral genome during replication (Verma et al., 2021). In the case of Zika virus (an enveloped positive strand RNA virus), interaction of the viral envelope (E) protein with multiple regions of Zika genomic RNA, [which includes two regions at the 5’- end (nt 135–294 and nt 734-899) and one region at the 3’- end (nt-10474- 10644)] is important for viral replication (Hou et al., 2017). The stem loop I (SL-I) in the 5’-UTR of Polio viruses interacts with host PCBP2 and viral proteinase-polymerase precursor protein 3CD to form a ternary complex that is important for viral RNA replication (Gamarnik and Andino, 2000). 5’-UTR of the Enterovirus 71 RNA interacts with the hnRNP K and hnRNP A1, which is important for viral translation and replication (Lin et al., 2008; Levengood et al., 2013). La protein interacts with both the 3’- and 5’-UTRs of CVB3 independently of the poly(A) tail, and seems to play a role in mediating cross-talk between the 5’- and 3’-ends of the CVB3 genomic RNA, facilitating viral RNA replication (Cheung et al., 2007). In coronaviruses, genomic and sub-genomic RNAs consist of 5’- and 3’-UTRs at their terminals and a transcriptional regulatory sequence (TRS) within the 5’-UTR. TRS helps in template switching during the synthesis of the negative-strand RNA by base pairing between the TRS-L and nascent TRS-BS by the viral transcriptase/replicase complex (Yang and Leibowitz, 2015).

#### 2.2.2 RNA-protein interactions during progeny virus assembly and release

Translation of genomic and sub-genomic RNAs produce non-structural and structural proteins, necessary for replication and progeny virus assembly, respectively. Replication of the viral genome produces multiple copies of itself, which need to be protected from host endonucleases and thus are compactly packaged inside the viral nucleocapsid shell. The capsid protein of the virus directly interacts with the viral genomic RNA and on its own or with the help of M protein (Membrane/Matrix protein in many RNA viruses), genomic RNA is packaged into the nucleocapsid shell. Progeny viruses are subsequently released out by exploiting the host cellular transport machinery. Host RBPs are involved in these steps as illustrated in the case of Flaviviruses (Diosa-Toro et al., 2020).

### 2.3 Impact of spatial and temporal binding of RBPs to the viral RNA

Localization of RBPs may be spatially restricted to specific intracellular organelles such as the ER, Golgi, lysosomes, recycling endosomes and autophagosomes. RBPs are also abundant in P-bodies and stress granules. RBPs may also be enriched at an intracellular site via RNA-protein/protein-protein interactions and liquid-liquid phase separation. Viruses may modulate the localization of RBPs or benefit from the presence of the RBP at a particular site. The ER and Golgi apparatus are essential for forming viral replication complexes and the biogenesis of viral membranes in many cases. HCV and DENV exploit ER-derived membranous webs, where RBPs like PTB stabilize viral RNA for replication (Anwar et al., 2009; Chatel-Chaix and Bartenschlager, 2014). Further, DENV 3’-UTR interacts with G3BP1/2 and DDX6, proteins found in stress granules and P-bodies, suggesting viral replication complexes localize between these granules (Ward et al., 2011). On the other hand, WNV disrupts P-body formation by recruiting DDX6 and other mRNA silencing components to viral replication sites, where they promote viral replication (Chahar et al., 2013). Lysosomes, endosomes, and autophagosomes are also key in viral entry, trafficking, replication, and survival. During SARS-CoV-2 infections, the autophagy receptor SQSTM1 (p62) interacts with the viral RNA, inhibiting autophagy and generating autophagosomes that serve as replication platforms (Kamel et al., 2021a). A 2021 study highlighted how autophagosomes containing DENV proteins and genomic RNA evade immune detection (Wu et al., 2021). Temporal regulation further complicates this process, with RBPs binding viral RNA at distinct stages of infection. For example, ChIRP-MS and qTUX-MS using SILAC labeling have provided insights into temporal changes in RNA interactions during SARS-CoV-2 and DENV infections (Viktorovskaya et al., 2016; Flynn et al., 2021).

## 3 Regulatory elements in the +ssRNA virus genome mediate its interaction with viral proteins and host proteins

**Figure 3:**
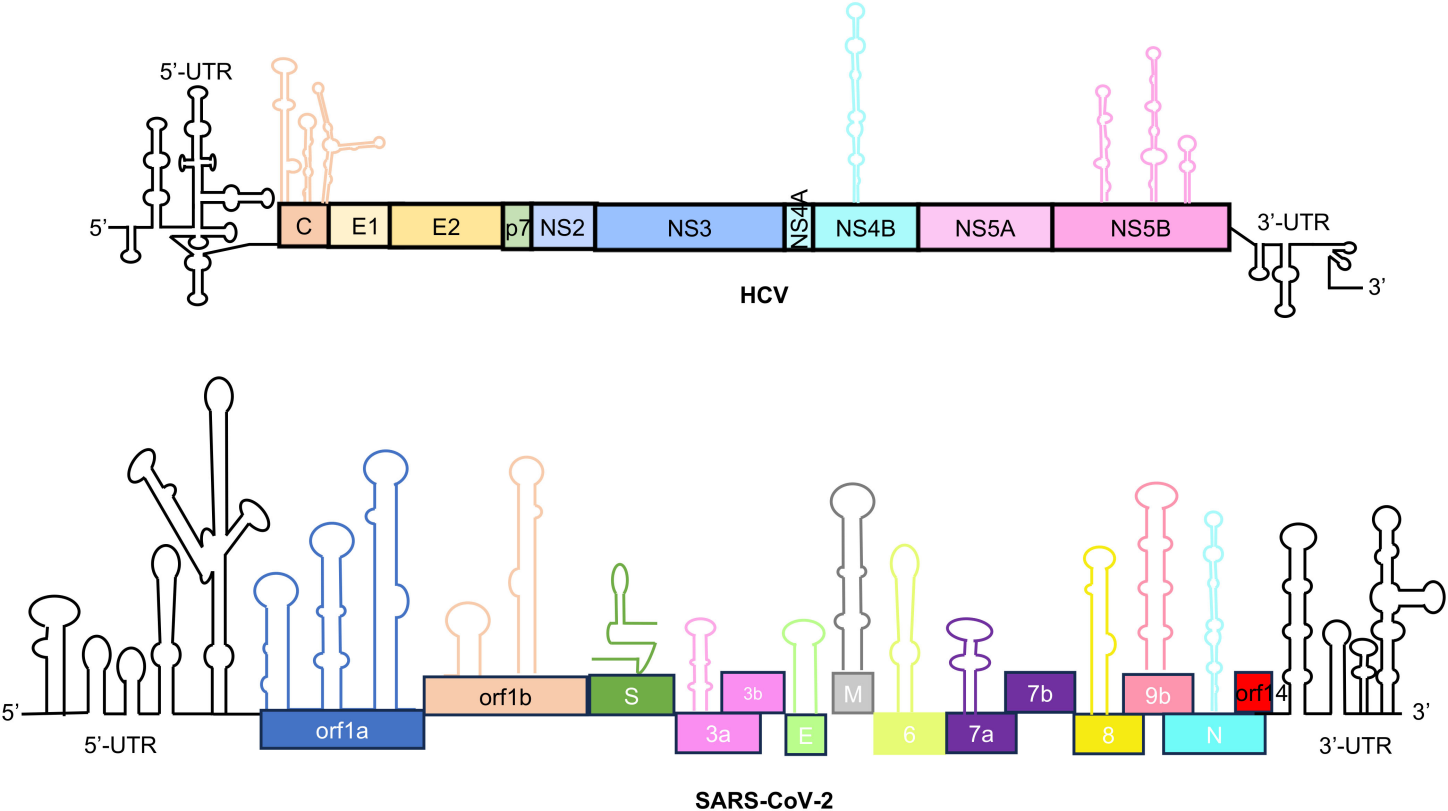
Schematic of the RNA regulatory elements present in the genome of HCV and SARS-CoV-2. Stem loops in the 5’ and 3’ UTR (untranslated region) regions have been indicated in black color. Stem loops present in internal region are represented against corresponding proteins. Top schematic is for Hepatitis C virus which includes - C, Core/capsid protein; E1 and E2, Envelope glycoproteins; p7, Viroporin; NS, non-structural proteins. The bottom schematic is for SARS-CoV-2 which includes – ORF 1a to ORF14, Open reading frame; S, Spike protein; E, Envelope protein; M, Membrane protein; N, Nucleocapsid protein. S, M, E, N are the structural proteins.

In contrast to DNA, RNA-RBP interaction is not necessarily sequence driven. Although there are well defined sequence motifs for recognition by specific RBPs, in many cases, RNA folds into secondary and tertiary structures generating specific conformations necessary for recognition by RBPs. Therefore, both sequence and structure of RNA regulatory elements are important for binding with RBPs. Regulatory elements are present at 5’-end, 3’-end and internal regions of the genome in +ssRNA viruses, schematically shown with examples of HCV and SARS-CoV-2 (**Figure 3**) (Tavares et al., 2021).

### 3.1 Regulatory elements at the 5’-UTR

5’-UTR of the +ssRNA viruses may be capped or uncapped. In the case of capped-RNA, UTR contains multiple stem loop (SL) structures, followed by Kozak sequence and initiation codon for the non-structural protein. Stem loops present in the 5’-UTR are important for protecting the RNA, assembling the translation initiation complex, and packaging the viral genome. They also aid in viral transcription and replication process. For example, 5’-UTR of SARS-CoV-2 spans 265 nucleotides and consists of 5 stem-loop structures. The transcriptional regulatory sequence (TRS) is present in the SL-III, which controls the discontinuous transcription (Liu et al., 2007; Sola et al., 2015; Miao et al., 2021). The SL-V has been indicated to be involved in viral RNA packaging and translation of ORF1ab polyprotein (Miao et al., 2021). In addition, both SL-III and SL-IV are targets for the binding of viral and cellular proteins, thus may play a role in viral replication (Sola et al., 2011; Madhugiri et al., 2016).

In the case of uncapped RNA, UTR contains multiple stem loop (SL) structures, followed by internal ribosome entry site (IRES) and initiation codon for the non-structural protein. IRES is a stretch of highly structured RNA elements, which directly recruit the initiation factors and promote translation through a scanning independent process, except type I IRES, which depends on the ribosomal scanning process. There are 5 major types of IRES based on their RNA structure and mode of ribosome recruitment. Notably, type I and type II IRES are found in the Picorna viruses such as PV and the FMDV, respectively. The PV IRES harbors six stem loops named as domain I to VI. The Domain I forms unique clover leaf structure and plays a critical role in replication of both the positive and the negative sense RNA. The domains II to VI are responsible for the PV IRES function. During PV infections, viral 2Apro cleaves the eIF4E binding N-terminal domain of the eIF4G without affecting its eIF3/eIF4A binding property. Stable association of the eIF4G with the PV IRES domain V enables its association with other initiation factors, leading to formation of the 43S preinitiation complex. The FMDV IRES is a classic example of the type II IRES. The domain IV of the FMDV IRES binds with scaffold protein eIF4G. The 3Cpro and Lpro of FMDV cleave the eIF4G. Importantly, the FMDV IRES skips ribosomal scanning, instead, IRES proximal stem loop formation brings 84 nucleotides downstream AUG, close to the first AUG to start the translation by direct ribosome transfer (Lee et al., 2017). The type III IRES is found in the 5’-UTR of the Hepatitis A virus genomic RNA (Brown et al., 1994). It requires eIF4E binding for translation initiation (Ali et al., 2001). The type IV IRES have been reported in the HCV (Hepatitis C virus)/HCV-like IRES. The 5’-UTR of HCV contains four domains: the domain I and II plays important roles in the viral replication while the domains III and IV are involved in translation (Khawaja et al., 2015; Kerr and Jan, 2016). The domains II and III contain several subdomains for interaction with the 40S ribosomal subunit. The type V IRES includes the long intergenic region (IGR) IRES, found between two open reading frames in the viral genomes and conserved in the dicistroviridae family. IGR IRES elements directly binds to the ribosomes and initiates translation with the alanine-tRNAi (ala-tRNAi) instead of the met-tRNAi, without involving the eIFs (Wilson et al., 2000; Pestova and Hellen, 2003). Thus, RNA-protein interactions play indispensable roles in the function of viral IRESs.

### 3.2 Regulatory elements at the 3’-UTR

3’-UTR of the +ssRNA virus genome usually contains a stretch of Adenine, followed by multiple SLs, which are important for binding of the viral RdRp and other virus-encoded and host factors as well as for RNA-RNA interactions. 3’-UTR is important for viral replication, translation and evasion of host antiviral response. For example, SARS-CoV-2-3’-UTR is 228 nucleotides long and contains 4 SLs. Pseudo-stem-loop (PK), bulge stem-loop (BSL), and S2M domain (HVR) in the 3’-UTR are supposed to be important for the life cycle of the virus (Rangan et al., 2020). The 3’-UTR carries distinct nucleotide combinations such as CTC, TGT, CGT for every group i.e., SARS-CoV-2, SARS-CoV, and Bat-CoVs, respectively. These nucleotide combinations overlap with S2m, a highly conserved RNA motif, which likely have a role in viral pathogenesis (Kelly et al., 2021). These positions were also found to overlap with BSL and PK regions of the 3’-UTR among all βCoVs. The hypervariable region consists of an octa-nucleotide sequence (5’-GGA AGA GG-3’) that is conserved among coronaviruses (Sola et al., 2011).

The coordinated interaction between 5’- and 3’-UTR through host and viral proteins forms the foundation for efficient replication of the virus (Nicholson and White, 2014). These long-range interactions create functional ribonucleoprotein complexes that enable three fundamental processes: genome cyclization, replication initiation and host immune modulation. The process begins with genome circularization, as exemplified by flaviviruses like DENV, where complementary sequences in the UTRs form panhandle structures that bring the RNA ends into proximity (Khromykh et al., 2001; Liu et al., 2020). This structural rearrangement is facilitated by host RNA-binding proteins such as DDX6, which specifically recognizes and stabilizes pseudoknot formations in the 3’-UTR to enhance both translation and replication efficiency (Liu et al., 2020). Similarly, in Hepatitis E virus, the 3’-UTR stem-loops SL1 and SL2 directly interact with the viral RdRp to initiate replication (Agrawal et al., 2001), while the 5’-UTR hairpin recruits the structural protein ORF2, likely for virion assembly (Surjit et al., 2004). Beyond structural roles, these terminal interactions serve as regulatory hubs. The polyadenylated 3’-UTR of HEV performs dual functions, serving as both a replication element and a potent activator of RIG-I-mediated innate immunity through its U-rich region (Sooryanarain et al., 2020). This exemplifies how viral RNA termini have evolved to balance replication needs with immune evasion strategies. The importance of host RBPs in maintaining these functional interactions is evident across virus families. Poliovirus employs hnRNP C as an RNA chaperone to keep its 3’-UTR in a single-stranded conformation optimal for replication initiation (Brunner et al., 2005; Ertel et al., 2010), while mouse hepatitis coronavirus utilizes PTB and hnRNP A1 to physically bridge its 5’ and 3’ UTRs (Li et al., 1999; Barton et al., 2001; Huang and Lai, 2001). Even in bacteriophage systems like Qβ, conserved mechanisms exist where internal-3’-UTR base-pairing, mediated by host factors, facilitates replicase assembly (Wu et al., 2009). From flavivirus genome cyclization to coronavirus UTR bridging, the conserved requirement for 5’-3’ communication mediated by specific RNA-protein interactions underscores their fundamental importance in the viral life cycle.

### 3.3 Cis-regulatory elements in the internal regions of the +ssRNA virus genome

Internal cis-regulatory elements refer to stable RNA secondary structures present in between the ORFs or in the coding region within the viral RNA. The presence of such cis-regulatory elements have been experimentally shown in the genome of +ssRNA viruses such as HCV and SARS-CoV-2 (**Figure 3**) (Tavares et al., 2021). For example, many cis-regulatory elements are found in the Core, NS4B and NS5B coding regions in the HCV genome (Tavares et al., 2021). Cis-regulatory elements are found in the ORF1a, ORF1b, S, ORF3a, E, M, ORF6, ORF7a/b, ORF8 and N coding region in the SARS-CoV-2 genome (Tavares et al., 2021). Further, nine TRS elements are present in the SARS-CoV-2 genome, which are important for sub-genomic RNA synthesis (Rangan et al., 2020). CRE is located in the 2C ORF of enteroviruses, the 2A ORF of species A rhinoviruses, the VP1 ORF of species B rhinoviruses, the VP2 ORF of species C rhinoviruses and cardioviruses, VP0 ORF of Parechovirus and upstream of the IRES in the FMDV (Mcknight and Lemon, 1998; Lobert et al., 1999; Goodfellow et al., 2000; Paul et al., 2000; Gerber et al., 2001; Mason et al., 2002; Al-Sunaidi et al., 2007; Cordey et al., 2008).

Another important internal regulatory element in the RNA virus genome is the frameshifting element. The frameshift element of SARS-CoV-1 has a pseudoknot (PK) structure. The dimerization domain of PK is critical for programmed ribosomal frameshifting (PRF), an essential event for forming ORF1a and ORF1b proteins from the same genomic region (Kelly et al., 2021). SARS-CoV-2 frameshifting element (FSE) is composed of a stem-loop attenuator, and a slippery sequence followed by a single-stranded spacer and an RNA pseudoknot. RNA-RNA interactions between the 3’-end of ORF1a and 5’-end of the ORF1b generates the FSE-arch, which is highly conserved among SARS-related coronaviruses and possess high folding stability *in vivo*. The FSE-arch likely controls the FSE activity (Ziv et al., 2020; Zhang et al., 2021).

## 4 Methods to generate RNA-protein interactome of +ssRNA viruses

### 4.1 Generation of RNA-protein interactome using biological samples

RNA-protein interactions can be experimentally demonstrated using either RNA-centric or protein-centric approaches. This review focuses on RNA-centric methods to identify the RBPs associated with the viral genomic or sub-genomic RNA. RNA-centric methods may be broadly classified into *in vitro* and *in vivo* methods. *In vivo* methods can detect the interaction between the whole viral genome or parts of viral genome and associated proteins whereas *in vitro* methods are generally used to detect the interaction between parts of viral genome and associated proteins.

#### 4.1.1 *In vitro* methods to detect RNA-protein interactions

**Figure 4:**
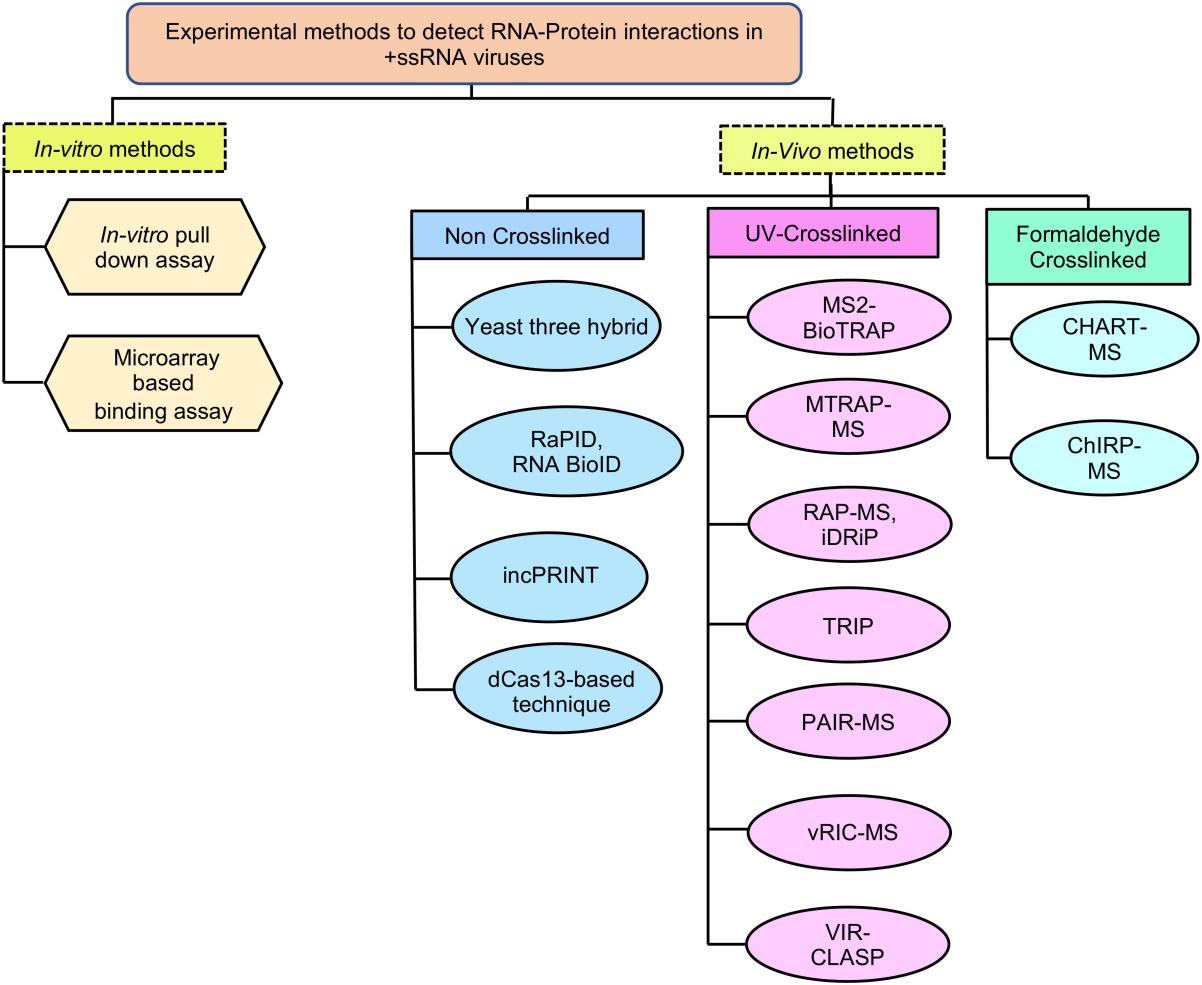
*In vitro and in vivo* methods to detect RNA-Protein interactions in +ssRNA viruses. RaPID, RNA-Protein Interaction Detection Assay; RNA BioID- RNA proximity biotinylation; incPRINT- In-cell protein-RNA interaction; MS2-BioTRAP, MS2-*in vivo* Biotin Tagged RNA Affinity Purification; MTRAP-MS, MS2-tagged RNA affinity purification and Mass spectrometry; RAP-MS, RNA Antisense Purification followed by Mass Spectrometry; iDRiP, Identification of Direct RNA-interacting Proteins; TRIP, Tandem RNA Isolation Procedure; PAIR, Peptide-Nucleic acid Assisted Identification followed by Mass Spectrometry; vRIC-MS, Viral RNA Interactome Capture followed by Mass Spectrometry; VIR-CLASP, Viral Cross-linking And Solid-phase Purification; CHART-MS, Capture Hybridization Analysis of RNA Targets followed by Mass Spectrometry; ChIRP-MS, Comprehensive identification of RNA-binding proteins by mass spectrometry.

*In-vitro* methods such as pull down and microarray-based binding assays are used to detect the interaction between parts of a viral genome and associated proteins (**Figure 4**). These methods are beneficial when the test RNA or protein is unstable, not expressed well *in vivo* or the RNA binding proteins are less abundant *in vivo*. *In vitro* assays are also useful in characterizing the molecular details of a particular RNA-protein interaction at nucleotide and amino acid level.

**Figure 5:**
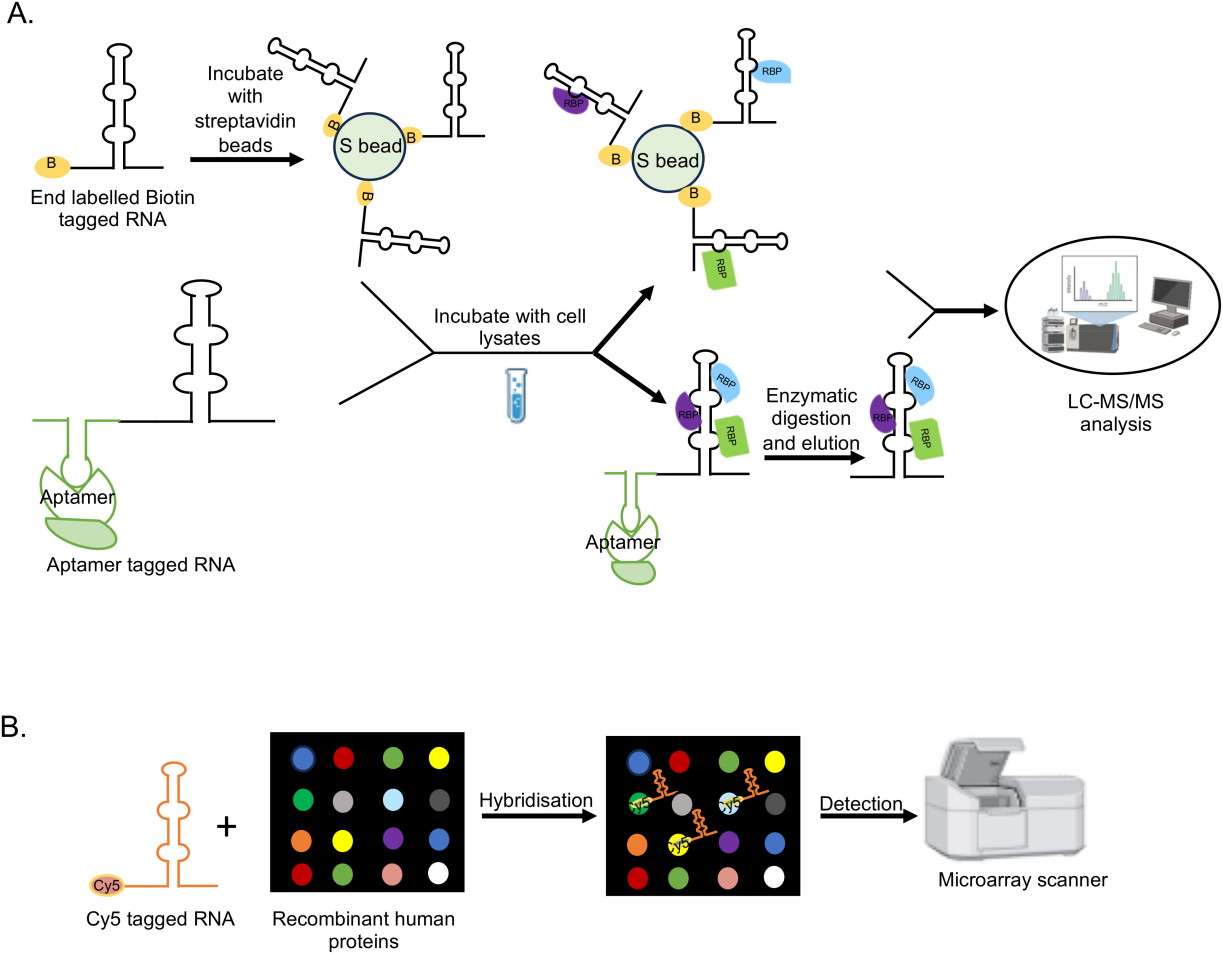
Schematic of *in vitro* methods to detect RNA-protein interactions. **(A)** *In-vitro* pull down assay. **(B)** Microarray-based binding assay.

In the case of pull down assay, the 5’- or 3’- end biotin-labeled RNA is synthesized *in vitro* and incubated with cellular extract, followed by isolation of the RNA-protein complex using streptavidin beads (Zheng et al., 2016). Alternatively, the RNA-protein complex may be isolated by using a biotin-labeled aptamer sequence against the test RNA (Srisawat and Engelke, 2001) (**Figure 5A**). In another study, Cys4 hairpin loop-tagged RNA has been used to select the test RNA bound complex, followed by elution of the test RNA-protein complex using imidazole, which activates the Cys4 endoribonuclease that cleaves the Cys4 RNA (Lee et al., 2013). The later technique may be useful in reducing the background signal as endogenously biotinylated proteins directly bind with the streptavidin beads irrespective of their RNA binding activity. RNA-protein complex may be UV crosslinked in an *in vitro* pull down assay. In a microarray-based binding assay, individual proteins are spotted on a microarray slide, followed by hybridization with a labeled test RNA (such as Cy5-labeled RNA) (Kretz et al., 2013) (**Figure 5B**). In both approaches, non-specific proteins are removed by multiple washing steps and interaction partners are detected by mass spectrometry or by fluorescence reading in the microarray scanner, respectively. Microarray-based binding assay detects direct interactions between the RNA and protein whereas pull down assay can detect both direct and indirect interaction partners. Although *in vitro* assays are simple and straight forward, it is limited by the fact that *in vitro* synthesized RNA may not fold properly or lack the native structure and modifications required for interaction with a particular interaction partner or protein complex. Further, High or low abundance of the protein(s) in the cellular extract may influence the result and proteins spotted on the microarray slides may not be properly folded or lack the required post-translational modification(s) or physiological environment required for interaction with the test RNA.

#### 4.1.2 *In vivo* methods to detect RNA-protein interactions

The limitations of the *in vitro* assays are partly resolved through *in vivo* methods, which offer physiological and functional advantages. Various technologies to detect RNA-protein interactions *in vivo*, with or without crosslinking of the complex are summarized (**Figure 4**). Although phase separation-based techniques for isolating RNA-protein complex have emerged to be a powerful approach to identify RBPs (Queiroz et al., 2019; Trendel et al., 2019; Urdaneta and Beckmann, 2020), this review will focus on techniques relevant to detection of viral RNA binding proteins.

##### 4.1.2.1 *In vivo* methods to detect RNA-protein interactions in non-crosslinked samples

**Figure 6:**
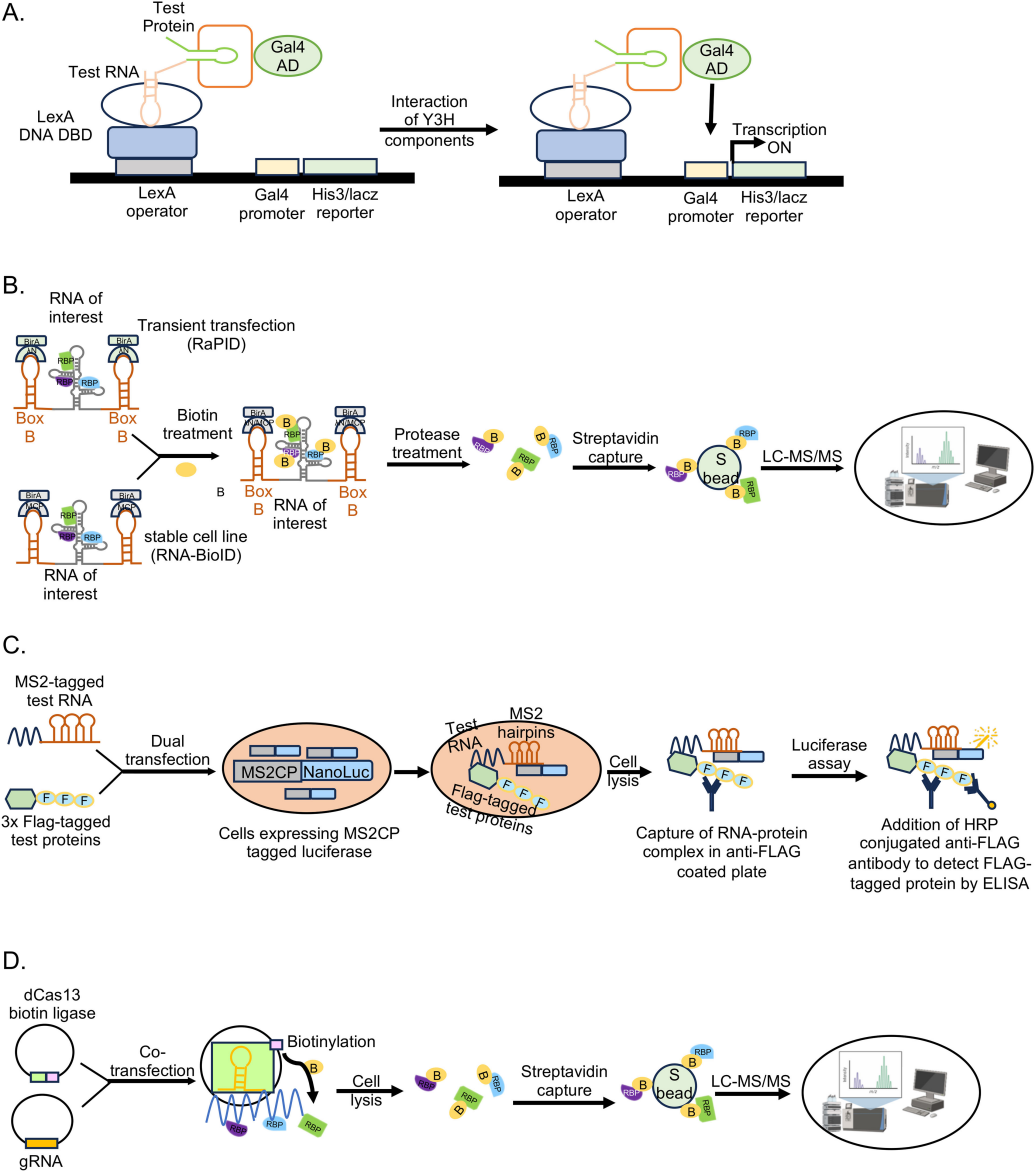
Non-crosslinked *in vivo* methodologies to study RNA-protein interactions. **(A)** Yeast three hybrid (Y3H) assay, **(B)** RaPID assay and RNA BioID, **(C)** incPRINT, **(D)** dCas13-based technique.

Among the crosslinking-independent *in vivo* techniques, Yeast three hybrid is a classical genetics technique, useful in detecting direct interaction between a test RNA and protein(s) in a physiological environment (SenGupta et al., 1996). Here, host proteins are expressed in the yeast cells using a cDNA expression library of the host cell type of interest as a fusion protein with the GAL4-AD (activation domain of the GAL4 transcription factor) (**Figure 6A**). Viral RNA is expressed as a fusion with MS2-binding RNA element at the 5’- or 3’-end. Specific interaction of viral RNA with a protein activates the *HIS3* (imidazoleglycerol-phosphate dehydratase) and *lacZ*(β-galactosidase) reporter genes, allowing growth of the yeast transformants in histidine deficient medium and colorimetric scoring by quantification of β-galactosidase activity, respectively. Interacting proteins are subsequently identified by isolating the cDNA clone and sequencing the plasmid DNA. Although the assay is conducted in a cellular milieu, which likely enables unbiased assessment of the interaction partners, screening of the cDNA library produces a lot of false positives and chances of misfolding of the test RNA and prey proteins cannot be ruled out.

Crosslinking-independent mammalian cell culture based proximity proteome labeling techniques such as RNA-protein interaction detection (RaPID) and the RNA proximity biotinylation (RNA BioID) have been reported (Ramanathan et al., 2018; Mukherjee et al., 2019; Verma et al., 2021; Kumar et al., 2023b). Proximity proteome labeling techniques rely on enzymes such as: Biotin ligases like BASU, BioID (mutant variant of the BirA enzyme) and its derivatives, which covalently attach biotin to proteins within 10-20nm radius; or ascorbic acid peroxidase (APEX) and its derivatives, which converts exogenously supplied biotin-phenol to biotin-phenoxyl radicals upon treatment with H_2_O_2_, resulting in covalent labeling of proteins. Both BASU and APEX label the proteins within 20nm radius, however APEX labeling is very fast (~1 min) compared to labeling by BASU (several hours) (Rhee et al., 2013; Paek et al., 2017; Samavarchi-Tehrani et al., 2020). Note that APEX labeling also requires treatment of cells with H_2_O_2._ Hence, choice of the proximity labeling enzyme is dependent on the experimental design.

RaPID assay identifies direct and indirect interaction partners of small RNA fragments (~132 nucleotides), which are expressed as chimeric RNA in fusion with an aptamer sequence such as Box B stem loop, which is recognized by the ΛN peptide (**Figure 6B**). A biotin ligase [BASU] fused to the ΛN peptide is recruited to the Box B, which biotinylates all proteins in its close proximity (~10-20nm range), including those associated with the RNA of interest. Biotinylated proteins are enriched and identified by LC-MS. RaPID assay has the advantage of detecting weak and transient RNA-protein interactions, however, the assay depends on overexpression of the test RNA. To overcome the limitation of overexpression of the test RNA and improve the efficiency of proximality labeling, Mukherjee et al., developed the RNA-BioID assay using genetically modified mouse embryonic fibroblasts (MEF). They used MEFs in which endogenous β-actin gene copies were replaced by β-actin with 24 MS2 binding sites (MBS) in their distal 3′-UTR and there was stable expression of a fusion of the nuclear localized signal (NLS), MS2 coat protein (MCP), GFP, and BirA* (MCP-GFP-BirA*) (Mukherjee et al., 2019). This approach identified a much higher number of interaction partners of the β-actin RNA, compared to other affinity-based methods.

Another crosslinking independent, RNA-tagging based *in vivo* technique is in-cell protein-RNA interaction (incPRINT). Here, the test protein is tagged with Flag epitope and the test RNA is tagged with MS2 stem loop sequence, which is expressed in cells along with MS2-coat protein fused to Luciferase. Test protein is captured from the cell lysate by Flag affinity beads, followed by Luciferase assay to detect its interaction with the test RNA (Graindorge et al., 2019) (**Figure 6C**). The assay may be scaled up to screen a library of Flag-tagged proteins against a test RNA.

Recent studies also demonstrated the utility of CRISPR-Cas targeting system in detecting RNA-protein interactions without crosslinking of the samples (Han et al., 2020; Lin et al., 2020; Yi et al., 2020; Zhang et al., 2020; Li et al., 2021). Using guide RNA (gRNA) specific to the test RNA along with a catalytically dead Cas13 (dCas13, dCasRx) fused to a biotin ligase (BASU, PUP-IT), it is possible to biotinylate proteins interacting with any endogenous RNA, which can be subsequently captured by streptavidin beads and identified by LC-MS (**Figure 6D**). Several modifications in the initial technique have been reported, which further improved the efficacy of the technique (Labun et al., 2019; Wessels et al., 2020). At the same time, more research is required to rule out the possibility of background noise due to off target binding by the gRNA. Note that, towards reducing the background noise in biotin ligase-based RNA-protein interaction detection techniques, recent studies have developed split biotin ligases, which gains enzymatic activity only when associated with the target RNA (Shekhawat and Ghosh, 2011; De Munter et al., 2017; Schopp et al., 2017; Cho et al., 2020).

##### 4.1.2.2 *In vivo* methods to detect RNA-protein interactions in crosslinked samples

Crosslinking of the RNA-protein complex *in vivo* arrests the interactions, which helps in capturing of weak and transient interactions. While crosslinking enhances the stability of the complex and increases the number of RBPs in the data set, there is a possibility of capturing nonspecific proteins due to over crosslinking and loss of bona fide RBPs due to inefficient crosslinking of weak interactions in a multiprotein complex. Both UV and formaldehyde are widely used in different techniques to crosslink the RNA-protein complexes *in vivo*. The choice of technique should be based on approximate information of the abundance of target proteins and test RNA, the strength of the RNA-protein interaction and size of the RNA-protein complex. It is noteworthy that although UV rays irreversibly crosslink nucleotide-protein interactions at zero distance via a covalent bond, it works less efficiently and weak interactions might be missed. On the other hand, overexposure to UV may have undesired consequences on the cellular processes. Hence, optimization of UV cross linking duration is important for success of the experiment. Formaldehyde reversibly crosslinks protein-protein, protein-DNA and protein-RNA interactions within 2A°, via covalent bond. However, formaldehyde crosslinking is less specific in capturing only RNA-protein interactions. Both RNA-tag and RNA hybridization based approaches have been used to detect RNA-protein interactions in crosslinked samples.

**Figure 7:**
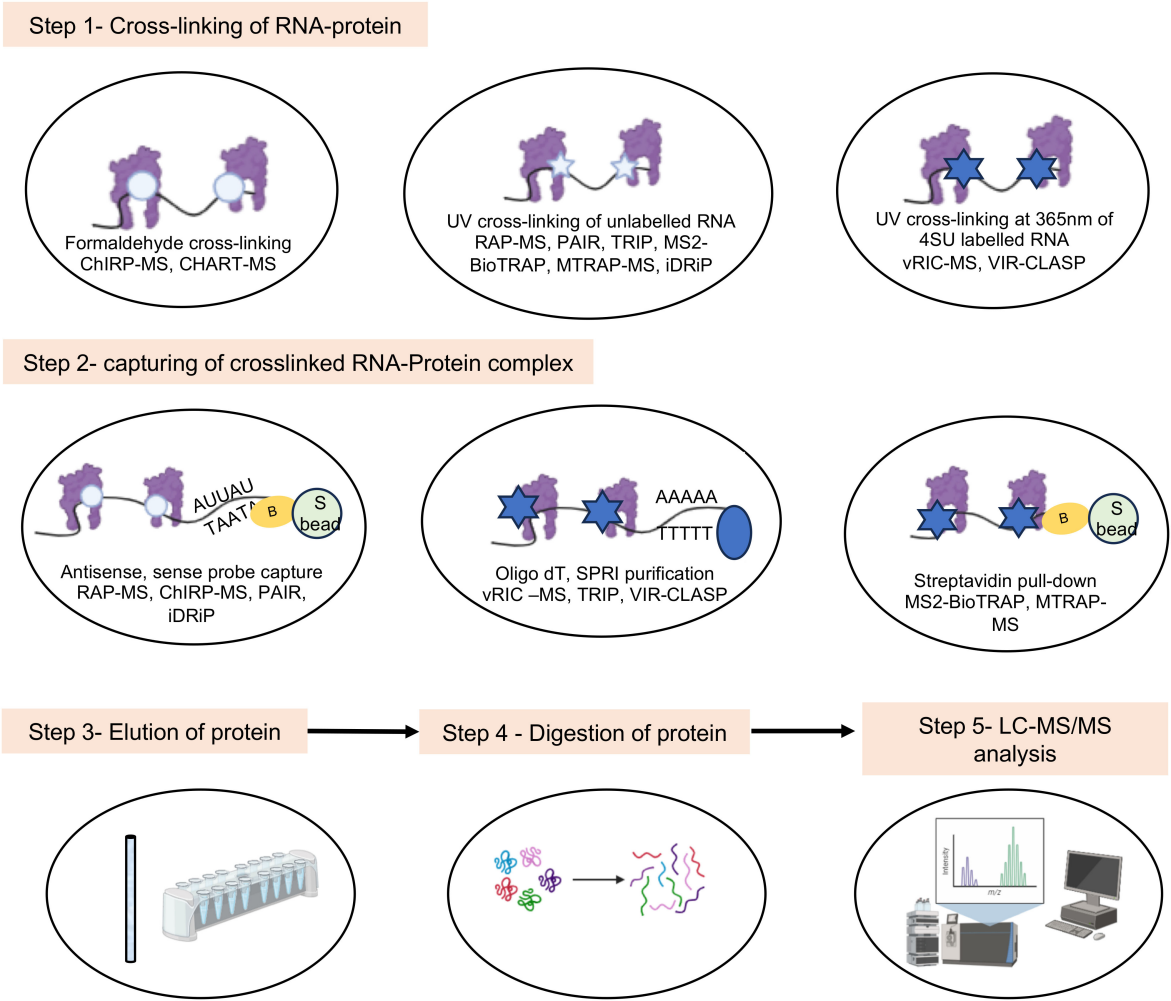
Schematic of major steps in the *in vivo* experimental methods involving mass spectrometry analysis to study viral RNA-protein interactions. White circle indicates formaldehyde crosslinking, white and Blue star indicate UV crosslinking, yellow circle indicates biotin tag.

RNA-tag based techniques depend on an aptamer sequence such as the MS2 stem loop element to capture the target RNA. RNA-protein interaction is stabilized by UV crosslinking and interacting proteins are revealed by pull down assay-LC-MS/MS. Techniques such as MS2-BioTrap, MS2-TRAP and MTRAP-MS are based on the above principle (**Figure 7**) (Tsai et al., 2011; Yoon et al., 2012; Liu et al., 2015).

RNA hybridization based approach have been employed in multiple techniques. Capture Hybridization Analysis of RNA Targets (CHART) and Comprehensive identification of RNA-binding proteins by mass spectrometry (ChIRP-MS) are two popular RNA hybridization-based techniques, in which formaldehyde is used to crosslink the samples (**Figure 7**) (West et al., 2014; Chu et al., 2015). Cells are treated with formaldehyde, followed by hybridization with biotinylated oligonucleotides (c-oligos). RNA-bound RBPs are purified using streptavidin beads, followed by protein identification by western blot or LC-MS/MS, for CHART and ChIRP, respectively. ChIRP was used to compare the RNA-protein interactome of SARS-CoV-2, Zika, and Ebola viruses (Flynn et al., 2021; Zhang et al., 2022). However, this technique is limited by the inefficiency of c-oligos to bind the different target loci with equal efficiency.

Compared to DNA based oligonucleotide probes used in CHART and ChIRP, antisense RNA based probes are more specific and expected to show less background noise. However, the use of RNA probes require more stringent experimental conditions due to inherently fragile characteristic of the RNA. RNA antisense purification (RAP), identification of Direct RNA-interacting Proteins (iDRiP) and tandem RNA Isolation Procedure (TRIP) are some notable techniques based on antisense RNA hybridization (**Figures 4**, **7**) (McHugh et al., 2015; Minajigi et al., 2015; Matia-González et al., 2017).

Further, Peptide-nucleic-acid (PNA) based probe has been used to detect RNA-protein interactions. In Peptide-nucleic-acid Assisted Identification (PAIR) assay, PNA is used to hybridize with the target RNA. PNA contains a photoactivable amino acid adduct, p-benzoyl phenylalanine (Bpa), which captures the nearby RBPs by photoactivated cross-linking. UV is used to covalently cross-link the PNA-Bpa with adjacent RBPs. Finally, PNA-RBP complexes are isolated using sense oligonucleotide magnetic beads, followed by LC-MS mediated identification of bound proteins (Zeng et al., 2006).

To address the low efficiency of UV cross linking, photoactivable ribonucleoside-enahnced (PAR) crosslinking techniques such as Viral RNA Interactome Capture (vRIC) and Viral cross-linking and solid-phase purification (VIR-CLASP) have been developed. Here, cellular RNA is metabolically labeled with 4-thiouridine (4SU), followed by UV cross-linking at 365nm (longer wavelength compared to conventional UV crosslinking at 254nm) and capture of the RNA-protein complex. After RNase digestion, quantitative proteomics is employed to reveal the captured RBPs (**Figure 7**). vRIC identified the RNA-protein interactome of SARS-CoV-2 and Sindbis virus (Kamel et al., 2021a; Kamel et al., 2021b). VIR-CLASP was used to identify the RNA-protein interactome of pre-replicated genome of the Chikungunya virus (Kim et al., 2020). Choice of the nucleoside analogue is decided based on its toxicity on the target cell and efficiency of its incorporation into the target RNA. A comparison of the advantages and limitations of different methods is summarized in **Table 1**.

**Table 1:** Advantages and limitations of notable methods to study RNA-protein interactions.

Technique	Advantages	Limitations
*In-vitro* methods	• Ability to use purified and/or well-defined RNA and protein components. • Both direct and indirect interaction can be detected. • Molecular details and biomolecular characteristics of the interactions can be easily analyzed. • Ability to detect the interaction between unstable RNA and proteins.	• Interactions obtained in such artificial condition may not be physiologically relevant. • It may not be possible to generate the *in vivo* structure of the test RNA and/or protein *in vitro*.
Yeast three hybrid	• Ability to unbiasedly detect the RNA-protein interactions between a test in a physiological environment. • Handy technique to characterize molecular details by mutagenesis.	• High false positive rate • Yeast may not be the ideal host for the test RNA-protein interactions.
RaPID, RNA BioID	• *In vivo* technique, independent of cross-linking of the test RNA-protein interactions. • Real time labeling of interacting proteins • Transient and stable interaction can be detected.	• Test RNA is not in its native state. • Length of RNA is restricted to around 200 nucleotides. • Endogenous biotinylated proteins need to be filtered out. • May not be possible to detect all RBPs present in a multiprotein complex.
lncPRINT	• Mass spectrometry-based analysis is not required to identify the proteins.	• A limited number of proteins can be identified.
dCas13-based technique	• Specificity of CRISPR (Clustered Regularly Interspaced Short Palindromic Repeats) technology extended to detect RNA-protein interactions.	• The design of effective gRNA is challenging. • Proximity-labeling enzyme biotinylates proteins even when it does not bind to RNA thus increasing non-specificity.
MS2-Bio Trap	• Test done in physiological condition. • High specificity due to UV-crosslinking.	• Test RNA is not in its native state. • Fusion of test RNA with MS2 RNA might alter its property.
RAP-MS iDRiP	• Detection of direct RNA-protein interactions. • Endogenous RNA-protein complex can be purified intact. • High specificity. • Test done in physiological condition.	• A high number of cells is required. • Technically demanding.
PAIR-MS	• Peptide-nucleic-acid (PNA) probe used. • High specificity. • Photoactivated cross-linking and UV cross linking.	• Synthesis of probe is costly.
vRIC-MS VIR-CLASP	• Entire viral RNA interactome can be captured. • High specificity. • Photoactivated cross-linking and UV cross linking.	• Selecting the right nucleoside analogue may be challenging due to its toxicity on the target cell and efficiency of its incorporation into the target RNA.
CHART-MS ChIRP-MS	• RNA-protein interaction are detected in a cellular environment. • Handling is easy due to the use of DNA based probe.	• Additional RNase H step is required to identify free sites for probes. • Inefficiency of C-oligos to bind the different target loci with an equal efficiency. • Formaldehyde cross linking is less specific. • DNA probes show high nonspecific signal.

### 4.2 Generation of RNA-protein interactome using predictive modeling

Predictive modeling relies on two essential components: algorithms and the data used to train them. In the case of RNA-protein prediction models, large-scale interaction data are required, typically generated through experimental means. Over the years, various databases containing such data have been established through wet lab experiments, literature mining, or computationally predicted interactions (**Table 2**). For instance, the ENCODE database contains eCLIP datasets from 223 experiments conducted in HepG2 and K562 cell lines, capturing interactions with 150 RBPs. This repository of extensive experimental evidence contributes to the robustness of the models, particularly as they rely on algorithms that require substantial data inputs. Using the above-mentioned source data, advanced computational tools have been developed to understand the intricacies of RNA-protein interactions and generate the RNA-protein interactome. Several studies have been dedicated to developing sophisticated algorithms that utilize sequence information to identify critical features indicative of RNA-protein binding affinity (Horlacher et al., 2023). The primitive approaches relied on sequence similarity, identifying patterns in sequences and their known interactions. While these models have low computational costs, they lack robustness. Understanding the fact that proteins adopt 3D structures before performing functions, alternative approaches leveraged upon structural information to provide a better understanding of potential affinities. Both methods have advanced significantly over time. However, fully deciphering structural information is complex due to the dynamic nature of proteins, making it challenging to comprehensively capture their true behavior. To address this, hybrid methods combining sequence and structure-based approaches have emerged to balance complexity and computational costs. A chronological list of such methods, along with their methodological descriptions, is listed (**Table 3**).

**Table 2:** Web-based resources for extracting RNA binding proteins, arranged in reverse chronology.

Method	Year	Description	Evidence	Reference
HydRA	2023	It is an ensemble RBP classifier, combines intermolecular protein interactions and internal protein sequence patterns, employing SVMs, CNNs, and Transformer-based models to predict RNA-binding capacity with high specificity and sensitivity.	Prediction	(Jin et al., 2023)
Pprint2	2023	It utilizes machine learning and deep learning for predicting RNA-interacting residues in proteins.	Prediction	(Patiyal et al., 2023)
RBPbind	2022	It predicts RNA-protein interaction probabilities, incorporating sequence specificity from RNAcompete experiments into Vienna RNA package recursions, enhancing accuracy.	Prediction	(Gaither et al., 2022)
RBPmap	2021	It uses sub-sequence that notably aligns with the RBP motif and additionally weaker matches surrounding the motif.	Prediction	(Paz et al., 2022)
RBPsuite	2020	It is a deep learning based method for predicting RBP binding sites on both linear and circular RNAs.	Prediction	(Pan et al., 2020)
RBinds	2020	It integrates RNA structure into networks for binding site prediction, with visualization and simulation tools.	Prediction	(Wang and Zhao, 2020)
ENCODE	2020	Experimental evidence in K562 and HepG2 cells through mapping and analysis of 1223 replicated datasets encompassing 356 RBPs.	Experimental	(Van Nostrand et al., 2020)
NPInter v4.0	2020	It hosts more than 600000 interaction of non-coding RNA with proteins.	Experimental	(Teng et al., 2020)
SMARTIV	2018	It predicts motifs from enriched k-mers, integrating information from ranked RNA sequences and their predicted secondary structure.	Prediction	(Polishchuk et al., 2018)
RNAct	2018	It provides an easy-to-use view of protein-RNA interactions in model organisms.	Experimental	(Lang et al., 2019)
omiXore	2017	It identifies RNA binding sites by discriminating interacting protein-RNA pairs using UV cross-linking data.	Prediction	(Armaos et al., 2017)
BindUP	2016	It predicts nucleic acid binding function using protein electrostatic features and structural properties, providing visualizations of electrostatic surface patches.	Prediction	(Paz et al., 2016)
CLIPdb	2015	It hosts 395 publicly available CLIP-seq data sets for 111 RBPs from four organisms: human, mouse, worm and Yeast.	Experimental	(Yang et al., 2015)
RAID	2014	It hosts more than 6100 RNA-associated interactions obtained by manually reviewing more than 2100 published papers.	Experimental	(Zhang et al., 2014)
PRIDB	2011	The Protein–RNA Interface Database (PRIDB) is a comprehensive database of protein–RNA interfaces extracted from complexes in the Protein Data Bank (PDB).	Experimental	(Lewis et al., 2010)
RBPDB	2010	It is a collection of experimental observations of RNA-binding sites, both *in vitro* and *in vivo*, manually curated from primary literature of four metazoan species (human, mouse, fly and worm).	Experimental	(Cook et al., 2010)

**Table 3:** Recent evolution of methods (from sequence-based to structural approaches) for predicting RNA-binding protein affinity.

Method	Year	Evidence	Architecture	Reference
BERT-RBP	2022	Sequence	Language model	(Yamada and Hamada, 2022)
DeepPN	2022	Sequence	CNN, GCN	(Zhang et al., 2022)
MultiRBP	2021	Sequence, structure	CNN	(Karin et al., 2021)
PRISMNet	2021	Sequence, structure	CNN	(Xu et al., 2023)
RNAprot	2021	Sequence, genomic annotation conservation	LSTM	(Uhl et al., 2021)
Multi-resBind	2021	Sequence, genomic annotation	CNN	(Zhao and Hamada, 2021)
RBP-ADDA	2021	Sequence	Adversarial domain adaptation	(Liu et al., 2022)
ResidualBind	2021	Sequence	CNN	(Koo et al., 2023)
kDeepBind	2021	Sequence	k-mer embedding CNN	(Tahir et al., 2021)
RBPSpot	2021	Sequence, structure	Embedding DNN	(Sharma et al., 2021)
DeepCLIP	2020	Sequence	CNN, BLSTM	(Grønning et al., 2020)
DeepRiPe	2020	Sequence, genomic annotation	CNN	(Ghanbari and Ohler, 2020)
RPI-NET/RNAonGraph	2020	Sequence, structure	GNN	(Yan et al., 2020)
DeepRKE	2020	Sequence, structure	Embedding, CNN, BLSTM	(Deng et al., 2020)
iDeepMV	2020	Sequence	Multi-view CNNs ensemble	(Yang et al., 2021)
DeepA-RBPBS	2020	Sequence, structure	CNN, biGRU	(Du et al., 2022)
MSC-GRU	2020	Sequence	CNN, biLSTM	(Shen et al., 2019)

**Figure 8:**
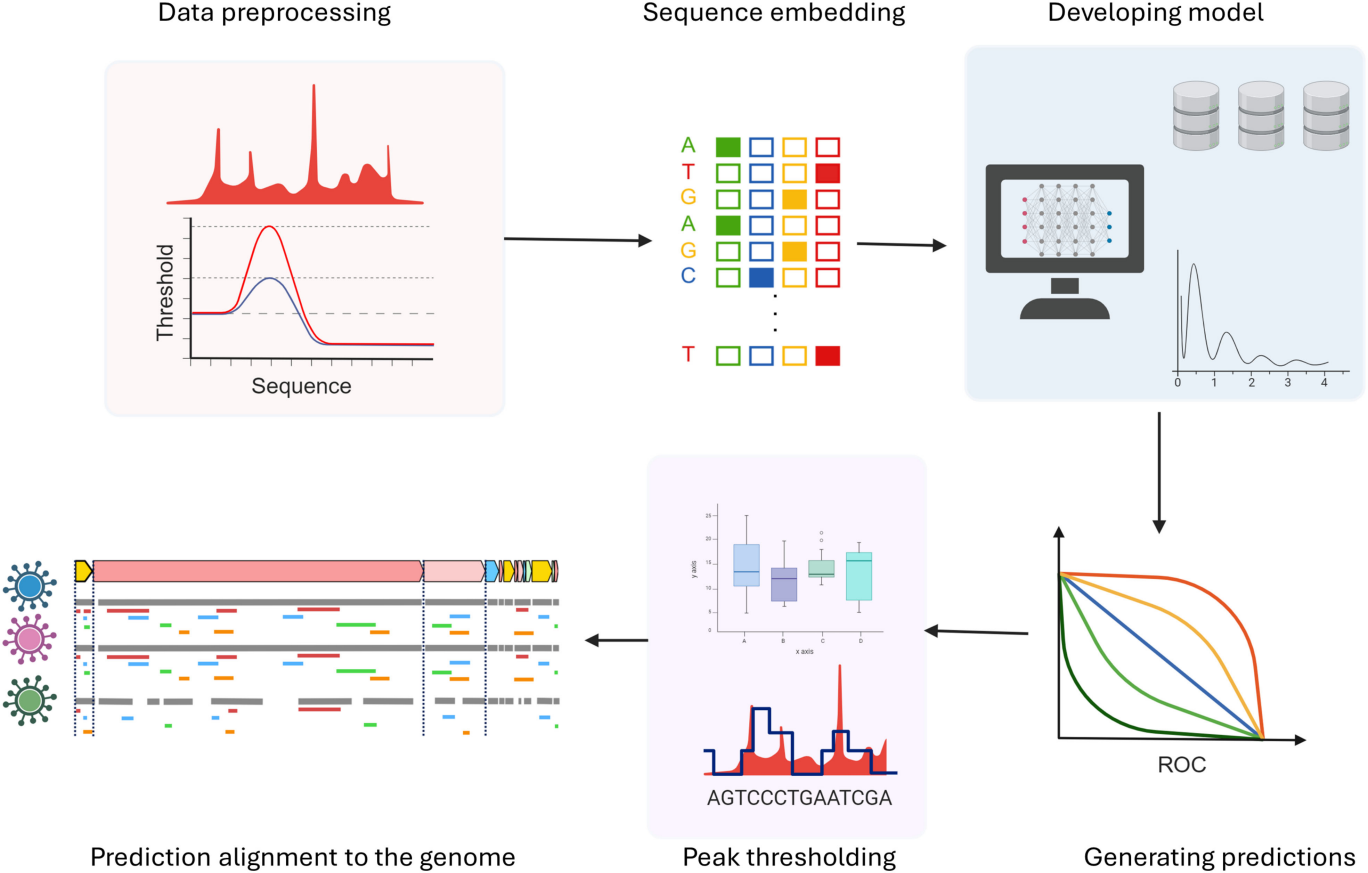
Schematic of computational methods to study RNA-protein interactions.

Although such algorithms are not exclusively designed to predict viral RNA and protein interactions, researchers have advanced these tools to predict inter-species interactions with promising results (Kazachenka et al., 2018). This endeavor involves a pipeline approach, employing established RBP interaction prediction algorithms (**Figure 8**). For algorithm training, datasets such as those from the ENCODE project has been used in this pipeline (Van Nostrand et al., 2020). First, the dataset undergoes pre-processing, wherein a defined window is established around each peak, facilitating the identification of potential binding sites for subsequent analysis. Later, this data is compared with peak information from RBPs used as controls, with a two-fold change along with statistical significance is considered. Sampling and randomization strategies are employed to mitigate false positives. Diverse algorithms, including recurrent neural networks (RNNs), extended short-term memory networks (LSTMs), and convolutional neural networks (CNNs) are utilized, employing supervised training methodologies with hyper-parameters such as sequence window size, algorithm layers, and learning rate. A negative sampling strategy is adopted for extracting sequence-level binding information, where the center of the window serves as the nucleotide of interest. Predictions are generated for randomized sequences, and a score is computed like a p-value. A high score indicates similarity in binding affinity between randomized and actual sequences, suggesting that binding may not be solely sequence or site-driven. This holistic approach yields a nuanced understanding of the RNA-protein interactome within the context of the entire genome.

### 4.3 Validation of the RNA-protein interactome data generated *in silico* or through Omics based technologies

Validation of the interactions between RBPs and their targets is crucial for distinguishing biologically meaningful associations from non-specific or background signals inherent in techniques like RNA-protein crosslinking or affinity purification. To minimize experimental noise, stringent controls such as mock immunoprecipitations (IPs), untagged viral RNA controls, or genetically modified cell lines with targeted RBP knockouts—are essential for establishing a reliable baseline. Mass spectrometry-based peptide identification platforms, including Mascot, MaxQuant, and FragPipe, enhance specificity by resolving ambiguous spectral data, while the CRAPome database helps systematically filter out common contaminants. Further, computational tools like Differential Enrichment analysis of Proteomics data (DEP), SAINTq, MIST score, and CompPASS, may be used, which apply stringent statistical criteria to enrich high-confidence interactors. A comprehensive computational pipeline processes raw affinity purification-mass spectrometry (AP-MS) data, performs quality control, and ranks biologically relevant bait-prey pairs across replicated experiments using these scoring methods (Verschueren et al., 2015). Post-processing filters such as false discovery rate (FDR) thresholds, fold-change cutoffs relative to controls, and consistency across replicates further help to eliminate spurious interactions.

In addition, an unbiased and independent method should be employed to reproduce the RNA-protein interactions identified in one method. For example, a combination of biochemical and imaging techniques are ideal for validating a subset of the interactome data. Super-resolution microscopy and advanced imaging techniques now allow real-time visualization of RBP-viral RNA dynamics within organelles, uncovering transient interactions previously missed. For example, the localization of HEV in recycling endosomes was studied using these imaging technologies (Bentaleb et al., 2022) and DENV and HCV replication and assembly were visualized using transmission electron microscopy (Chatel-Chaix and Bartenschlager, 2014). These methods are robust and broadly applicable across +ss RNA viruses, providing valuable insights into how different viruses manage viral RNA within cells, and identifying conserved or distinct mechanisms for potential antiviral targets.

RNA-SELEX (Systematic Evolution of Ligands by Exponential Enrichment) has emerged as a powerful tool to identify the specific RNA targets through iterative rounds of selection and amplification (Ellington and Szostak, 1990; Tuerk and Gold, 1990). SELEX has been used for determining the binding site of a protein on RNA (Manley, 2013). RNA-based Capture-SELEX has been used for selecting small molecule-binding aptamers (Ye and Jankowsky, 2020). Analogous to SELEX, another notable method, named massively parallel RNA assay combined with immunoprecipitation (MPRNA-IP) has been developed for high-throughput analysis of RNA–protein interactions *in vivo* (Lee et al., 2024). These methods are useful for improving the accuracy of molecular characterization and validation of the RNA-protein interactions.

Importantly, vast datasets generated in SELEX have expanded the scope of machine learning models by incorporating information about the intermediate interaction steps, in contrast to traditional machine learning (ML) models, which often rely on the final binding information, overlooking the iterative modification in interaction. ML models can learn patterns from SELEX data to predict which RNA sequences will likely bind a given protein with high affinity. It can also help identify sequence motifs, secondary structures, or physicochemical properties important for binding. Databases like HTPSELEX has been developed for training models and tools like DeepPBS (a geometric deep-learning model), GraphProt, BindSpace have been developed to predict RNA-protein binding (Maticzka et al., 2014; Yuan et al., 2019; Mitra et al., 2024). These advanced methods have leveraged the richness of SELEX datasets.

## 5 Importance of RNA-protein interactome of viruses in decoding the viral life cycle and antiviral discovery

It is important to evaluate the functional significance of the RNA-protein interactions to understand the molecular details of the viral life cycle and identify new targets for antiviral development. Suitable experimentally amenable tools such as non-infectious replicon of the virus or infectious/attenuated virus strains are useful resources for such studies.

### 5.1 Approaches to decode the life cycle of +ssRNA viruses using the viral RNA-protein interactome dataset

The RNA-protein interactome of a +ssRNA virus constitutes a set of proteins that directly or indirectly associate with the viral genome. These proteins need to be prudently analyzed to interpret and extrapolate their biological functions in the infected cells. This information forms the basis to hypothesize a mechanism of viral life cycle and pathogenesis, which is subsequently evaluated by suitable experimental models. Enrichment analysis has emerged as a standard approach to analyze large gene lists to produce a data-driven information that is easier to interpret. This analysis involves statistical testing of pathways and processes for over-representation in the experimental gene list compared to what would be expected by chance. Several common statistical tests are utilized, considering factors such as the number of genes detected in the experiment, their relative rankings, and the number of annotated genes. Some well-known web-based applications for such analysis include the Kegg pathway, Reactome pathway, GSEA (gene set enrichment analysis), Panther, and Gene Ontology (Subramanian et al., 2005; Thomas et al., 2022; Aleksander et al., 2023; Kanehisa et al., 2023; Milacic et al., 2024). These tools facilitate the identification of key pathways, functions and processes that are highly influenced by the identified set of genes. Moreover, RBP2GO and the RBP Image Database play crucial roles in elucidating the role of RBPs in viral infections (Caudron-Herger et al., 2021; Benoit Bouvrette et al., 2023). RBP2GO and the RBP Image Databases provide ontological information about the functions, processes, and cellular locations of RBPs, shedding light on their involvement in viral replication, RNA processing, and host immune responses. Once a hypothesis is formulated based on the acquired knowledge, appropriate experimental models are designed to validate the predictions.

### 5.2 Methods to unlock the therapeutic potential of RNA-protein interactome of +ssRNA viruses

As mentioned above, functional analysis of RNA-protein interactome data provides significant insight into the life cycle and pathogenesis of the corresponding virus. Such intricate understanding of viral lifecycle helps to identify and experimentally validate potential antiviral targets. Both the interactome data and the antiviral targets may be considered for screening antiviral drugs. Computational or experimental model-based screening methods may be followed to identify antiviral drugs either by *de novo* [identification of antiviral potential of a new chemical entity (NCE)] or drug repurposing [identification of a new therapeutic application of an existing drug] approach. *De novo* drug discovery is extremely expensive and time consuming whereas the drug repurposing strategy holds the potential of immediate therapeutic impact at a much lower cost. The main advantage of repurposed drugs is attributed to their prequalification through safety and toxicity tests in preclinical and human trials. Multiple computational methods may be pursued to discover antiviral drugs. Once a drug candidate is identified, it should be validated using wet lab experiments before proceeding with preclinical studies.

#### 5.2.1 Computational methods for capturing drug targets

The *in silico* drug discovery pipeline begins with target identification, which is very challenging, as proteins may require one or more interaction partners to execute essential functions. To address these limitations, different algorithms are employed to prioritize network nodes/proteins based on sensitivity or their potential to induce phenotypic changes. Some notable tools include CaNDis, CytoHUBBA, and NetEPD (**Table 4**). Another important aspect is identifying proteins that can control the information flow in the network, which can be obtained using tools like konnect2prot and NetControl4BioMed (**Table 4**). Sometimes, we are also interested in exploring proteins with similar functions to known therapeutic candidates for various reasons, such as being non-targetable or crucial for the system. In such cases, we can use guilt-by-association-based methods like Netpredictor to identify proteins with similar functions.

**Table 4:** Useful web-based resources for antiviral discovery (chronologically sorted).

Name	Year	Description	Reference
cytoHUBBA	2014	It is a Cytoscape plugin offering diverse topological analysis methods for ranking network nodes, with the newly proposed “maximum neighborhood component” (MCC), demonstrating superior precision in predicting essential proteins from the Yeast PPI network.	(Chin et al., 2014)
Netpredictor	2018	R package for prediction of missing links in any given unipartite or bipartite network using random walk restart algorithm.	(Seal and Wild, 2018)
NetEPD	2020	It is a web service integrating diverse databases and computational methods for accurate prediction of essential proteins.	(Zhang et al., 2020)
CaNDis	2021	It is a server for exploring a human causal interaction network, expanded with disease and FDA-approved drug data to construct a disease-disease network.	(Škrlj et al., 2021)
NetControl4BioMed	2021	It generates directed protein-protein interaction networks and used in controllability analysis of target structural network.	(Popescu et al., 2021)
Konnect2prot	2022	Build a functional network with context specificity and identifies important proteins that spread information in the network.	(Kumar et al., 2023a)

Later, the identified target needs to be modulated (activated or inhibited), which often requires small molecules, due to their various pharmacokinetic properties. These molecules could be newly synthesized or already available drugs. Such choices are made based on factors like time, cost, availability etc.

#### 5.2.2 Data driven screening methods for small molecule identification

The data-driven drug discovery process depends on online resources, including clinically oriented drug databases (for example, PharmGKB and RxList) and chemically oriented drug databases (for example, Zinc, TTD and PubChem). While clinically oriented drug databases provide in-depth clinical information, chemically oriented drug databases generally provide nomenclature and structural properties of the compounds (**Table 5**).

**Table 5:** Online drug databases and tools for antiviral discovery.

Name	Year	Description	Reference
(A) Clinically oriented drug databases			
PharmGKB	2000	It serves as an openly accessible online repository dedicated to consolidating, curating, integrating, and disseminating information pertaining to the influence of human genetic variability on drug response.	(Whirl-Carrillo et al., 2021)
RxList	1995	It is an online medical resource dedicated to offering detailed and current pharmaceutical information on brand and generic drugs.	(RxList)
(B) Chemically oriented drug database			
TTD	2014	The Therapeutic Target Database (TTD) systematically collects nine categories of established druggability characteristics for a wide range of targets, aiding in the identification and validation of innovative drug targets. Top of Form	(Zhou et al., 2024)
ChemSpider	2007	This database houses data on a vast array of molecules, exceeding 100 million, aggregated from over 270 distinct sources. Each molecule is assigned a unique identifier, known as the ChemSpider Identifier.	(Pence and Williams, 2010)
PubChem	2004	It has an extensive collection of over 293 million substance descriptions, featuring 111 million distinct chemical structures and encompassing 271 million bioactivity data points derived from approximately 1.2 million biological assay experiments.	(Kim et al., 2023)
ZINC	2004	It hosts a library of more than 230 million readily available compounds in 3D formats for docking purposes. Additionally, it offers access to over 750 million purchasable compounds, facilitating rapid analog searches within a minute.	(Sterling and Irwin, 2015)
(C) Online drug discovery tools			
Drug Central	2016	It is a publicly accessible drug information resource that includes 4950 drugs and related additional data sources such as FDA Adverse Event Reporting System (FAERS) and L1000 gene perturbation profile distance matrices.	(Avram et al., 2023)
ChEBI	2009	This database compiles data on compound bioactivity against drug targets, with bioactivity measurements reported in Ki, Kd, IC50, and EC50 values.	(Degtyarenko et al., 2007)
STITCH	2008	Search Tool for Interacting Chemicals (STITCH) consolidates dispersed data on protein-small molecule interactions into a single, user-friendly resource, now expanded to cover 430,000 chemicals. The latest release introduces a network view showcasing binding affinities, facilitating quick insights into chemical effects on interaction partners.	(Szklarczyk et al., 2016)
DrugBank	2006	The latest version of the database contains 4563 FDA approved drugs and 6231 investigational drugs, along with information on drug-drug and drug-food interactions.	(Wishart et al., 2008)

Both these databases have been used by several laboratories as source data for developing drug discovery tools. For example, Drug Bank was developed by combining the attributes of clinically and chemically oriented drug databases to serve as a handy yet comprehensive tool to search drug molecules and get details of their sequence, structure, mode of action, targets as well as biological or physiological consequences of drug action (Knox et al., 2024). Drug central is a platform on similar line (Avram et al., 2023). ChEMBL is another notable online tool that consolidates the bioactivity information of drugs (Zdrazil et al., 2024).

#### 5.2.3 *In silico* methods for discovery of small molecules: structural and machine learning approach

It is a proven fact that the proteins are dynamic in nature and so targeting a static snapshot may not be a very comprehensive approach. Therefore, multiple computational tools have been developed to virtually decipher the three-dimensional (3D) structure of biological molecules in their functionally active state and analyze the interaction among different biological molecules such as RNA-protein interactions, protein-drug interaction and RNA-drug interaction. The hallmark of computational structural study is its ability to generate and analyze multiple interconverting states by studying its thermodynamic properties. Molecular docking and molecular dynamics (MD) simulation techniques are used to characterize the complexities of RBP-drug interactions. Additional methods such as advanced quantum mechanics/molecular mechanics computation, Martini coarse-grained force field molecular modeling and Elastic network models may be adapted to analyze RNA-protein-drug interactions (Monticelli et al., 2008; Pokorna et al., 2018). Further, quantitative structure-activity relationship (QSAR) modeling have played a significant role in computer-aided designing of drug molecules. Recent advances in high-performance computing (HPC) and artificial intelligence (AI) technologies have propelled the transition of QSAR to deep QSAR (a combination of QSAR, more complex statistics and machine learning techniques), which is more robust in structure-based virtual screening (Gini et al., 2019; Selvaraj et al., 2023). Both clinical and chemically oriented drug databases may be used in this approach.

*In silico* analysis offers the advantage of simultaneous analysis and optimization of 3D structure of the interaction partners as well as drug molecules, which significantly expedites the optimization process. However, such an approach has inherent limitations such as the requirement of high computing power, lack of knowledge regarding 3D structural details of many biological molecules, inability to integrate biological information, and high false positive rate of molecular dynamic simulation analysis. Some of the above-mentioned limitations have been resolved by developing the computational analysis of novel drug opportunities (CANDO) platform (Minie et al., 2014). CANDO is a model independent approach to drug discovery. It leverages the evolutionary basis of protein and small molecule interactions and also considers the known biological data about interaction partners. Importantly, all the analyses do not require high computational power. CANDO platform has been used to identify several drug candidates against COVID-19 (Mangione et al., 2022).

#### 5.2.4 Experimental validation of drug candidates

After identifying a suitable antiviral target in the RNA-protein interactome data and *in silico* screening of drug libraries shortlists potential antiviral candidates, which may be evaluated through suitable wet lab-based assays. If a known drug molecule is identified, its antiviral potential against the corresponding virus may be directly evaluated. Alternatively, small molecule libraries of NCEs (new chemical entities) or FDA (Federal Drug Agency, USA) approved drugs may be screened against a specific target using appropriately validated assays. Potential drug molecules may be characterized by NMR (nuclear magnetic resonance), X-ray-crystallography and structural mass spectrometry (Britt et al., 2021). Once the antiviral potential of the drug molecule is evaluated in cell-based models and small animal models (if available), its efficacy may be evaluated through subsequent pre-clinical studies and clinical trials.

## 6 Conclusion

A thorough understanding of the RNA-protein interactions prevalent in the life of +ssRNA virus is fundamental to decoding the life cycle and mechanism of viral pathogenesis, knowledge of which is essential for developing specific antivirals. Recent studies have revealed the RNA-protein interactome of a few +ssRNA viruses such as SARS-CoV-2, Dengue Virus and Zika virus. These studies have demonstrated the power and functional utility of omics-based technologies in interrogating the RNA-protein interactome of +ssRNA viruses and provided convincing proof regarding the value of such technologies in gaining deeper insight into the life cycle and pathogenesis mechanism of +ssRNA viruses. Future research should aim at developing more sophisticated and advanced methods, including kinetic models. Further, considering the dynamic nature of the interaction between the RNA, protein and drug, differential equation-based mathematical models should be useful in characterizing them. Finally, the development of more efficient X-ray-crystallography and structural mass spectrometry methods should help in the antiviral discovery process. By coupling the data identified from RNA-protein interactome analysis with the drug discovery pipeline, it should be possible to develop potent antivirals against pathogenic +ssRNA viruses of medical importance.
